# Bio-Polyethylene-Based Composites Reinforced with Alkali and Palmitoyl Chloride-Treated Coffee Silverskin

**DOI:** 10.3390/molecules24173113

**Published:** 2019-08-27

**Authors:** Franco Dominici, Daniel García García, Vicent Fombuena, Francesca Luzi, Debora Puglia, Luigi Torre, Rafael Balart

**Affiliations:** 1 Civil and Environmental Engineering Department, University of Perugia, Strada di Pentima 4, 05100 Terni, Italy; 2 Instituto de Tecnología de Materiales (ITM), Universitat Politècnica de València, Plaza Ferrandiz y Carbonell 1, 03801 Alcoy, Alicante, Spain

**Keywords:** coffee silverskin, biopolyethelene, alkali, palmitoyl chloride, composites

## Abstract

This work investigates the feasibility of using coffee silverskin (CSS) as a reinforcing agent in biobased polyethylene (BioPE) composites, by adding it in bulk and thin film samples. The effect of two different treatments, alkali bleaching (CSS_A) and esterification with palmitoyl chloride (CSS_P), on mechanical, thermal, morphological and water absorption behavior of produced materials at different CSS loading (10, 20 and 30 wt %) was investigated. A reactive graft copolymerization of BioPE with maleic anhydride was considered in the case of alkali treated CSS. It was found that, when introduced in bulk samples, improvement in the elastic modulus and a reduction in strain at maximum stress were observed with the increase in CSS fraction for the untreated and treated CSS composites, while the low aspect ratio of the CSS particles and their poor adhesion with the polymeric matrix were responsible for reduced ductility in films, decreasing crystallinity values and reduction of elastic moduli. When CSS_A and CSS_P are introduced in the matrix, a substantial reduction in the water uptake is also obtained in films, mainly due to presence of maleated PE, that builds up some interactions to eliminate the amounts of OH groups and hydrophobized CSS, due to the weakened absorption capacity of the functionalized CSS.

## 1. Introduction

In the last years, a strong increase in environmental concerns has arisen. This fact, together with the continuous increase in petroleum prices and the overall depletion of fossil fuels have led to intensive research on the development of environmentally friendly materials [1]. One of the engineering fields that have experienced a more valuable growth is that related to composite materials with natural fillers/reinforcements such as natural fiber reinforced plastics (NFRP) and wood plastic composites (WPC) [2]. The use of natural fillers/reinforcements into polymeric matrices could lead to multiple advantages, such as clear cost reduction, lightness and good balance on mechanical properties, and a marked low environmental impact as well, due to the use of renewable materials [3]. For these reasons some technological sectors such as aerospace, automotive, building among others and other sectors, such as packaging, have shown a clear interest on these materials [4,5,6]. Despite this, natural fillers/reinforcements are majorly lignocellulosic materials and, therefore, they show an extremely high hydrophilicity. This results in poor adhesion with the usually hydrophobic polymer matrix. In addition, the hydrophilic nature of the filler/reinforcement also leads to aggregate formation (poor particle dispersion), which results in poor material cohesion and, subsequently, poor mechanical performance [7,8]. Improvement of interface adhesion in NFRP or WPC can be achieved by several strategies: on one hand, by lowering the hydrophilicity of the filler/reinforcement and on the other hand, by promoting chemical interactions between the filler/reinforcement and the polymer matrix. To achieve this objective several approaches can be used, such as selective surface modification of fillers, modification of the polymer matrix by additives and using compatibilizers to provide increased interactions among the polymer-particle interface [9]. A typical matrix modification is graft copolymerization of the base polymer with maleic anhydride (MA), which can be obtained by reactive extrusion [10,11,12,13]. During this process, an initiator promotes free radical formation on the polymer matrix chains and, subsequently, these unstable free radicals can react with the unsaturation contained in MA thus leading to graft MA into polymer chains. The attached anhydride groups can react with hydroxyl groups in the lignocellulosic filler/reinforcement to form ester bonds which, in turn, have a positive effect on increased adhesion among polymer-particle [14]. Some examples of selective modification of lignocellulosic fillers and/or reinforcements include mercerization, acetylation, benzoylation, peroxide treatment and the use of coupling agents with or without heat [15]. Nevertheless, recent studies have demonstrated that treatment of lignocellulosic wastes with fatty acid derivatives (oleoyl chloride, palmitoyl chloride, among others) is an effective way to selectively modify their surface with an important increase in hydrophobicity, which, in turn has a positive effect on increased polymer-particle adhesion. Fatty acid salts can react with hydroxyl groups contained in cellulose and hemicelluloses by esterification, leading to a remarkable increase in hydrophobicity. Garcia-Garcia et al. [16] investigated the effect of a hydrophobic treatment with palmitoyl chloride on spent coffee ground (SCG) powder and compared it with conventional treatments based on silanization with (3-glycidyloxypropyl) trimethoxysilane and the use of a maleinized copolymer compatibilizer (PP-g-MA) in terms of mechanical, morphological and thermal properties, as well as the effects on water absorption of polypropylene/SCG composites. The results showed increased hydrophobicity on SCG by treatment with palmitoyl chloride, leading to a composite with balanced mechanical and thermal properties. Corrales et al. [17] studied the effect of the chemical modification of jute fibers with oleoyl chloride with the subsequent increase in hydrophobicity.

Industrial wastes and agricultural by-products represent an excellent alternative to wood and natural fibers to be used as reinforcements in composite materials, due to their availability, abundance and, especially, their low cost [18]. The coffee industry is, with difference, one of the industries with most waste and by-product generation, since coffee is the third most consumed drink after water and tea and the second largest traded commodity after petroleum. As a consequence of this big market, this industry generates huge amounts of wastes and by-products such as husks, hulls, defective beans, coffee silverskin, and spent coffee grounds during the pulping, curing, roasting, and brewing of coffee [19]. Coffee silverskin (CSS) is a thin tegument contained in the external layer of the green coffee beans, and represents, approximately 4.2 wt % of the beans. CSS is the main by-product obtained after coffee beam roasting and has no commercial value [20], so that, it usually finds its end-of-life in controlled landfills or it is subjected to incineration which is a source of contamination, due to its high organic content and presence of chemical compounds such as caffeine, tannins and polyphenols [21]. Despite this, this waste has recently attracted some interest as a source of bioactive compounds for the food industry [22,23,24], cosmetics and beauty products [25,26], or pharmaceutical industry [27] due to its high content on dietary fiber, phenolic compounds or antioxidant, such as melanoidins. Nevertheless, this is not the best solution in terms of the added value, by taking into account the huge amounts of this waste, around 2 billion tons per year [28].

A potential alternative to minimize this huge amount of CSS waste is its use as filler/reinforcement in composite materials. Sarasini et al. [29,30] studied the effect of different loadings of CSS as-supplied state and also after a grinding and sieving process in biodegradable composites with poly(butylene adipate-co-terephthalate)–PBAT/poly(3-hydroxybutyrate-co- 3-hydroxyvalerate)–PHBV blends. They reported an increase in the tensile modulus with the CSS content without compromising the strength, and a positive increase in the Vicat softening temperature (VST) and crystallinity degree. They also studied the effect of different compatibilizers, a bio-sourced maleinized linseed oil (MLO) and a traditional silane, namely (3-aminopropyl)- triethoxysilane) (APTES) on mechanical and thermal behavior of these composites. Zarrinbakhsh et al. [31] studied the effect of the addition of 25% CSS into a PP matrix. They reported an increase in the tensile and flexural modulus, as well as an increase in the impact-absorbed energy and the heat deflection temperature (HDT) with regard to neat PP.

The main aim of this study was to investigate the effect of two different treatments for coffee silverskin (CSS), alkali bleaching and esterification with palmitoyl chloride, on mechanical, thermal, morphological and water absorption behavior of composites (bulk and film) based on a bio-based poly(ethylene) (BioPE) and different CSS loading (10, 20 and 30 wt %). In the case of composites containing alkali treated CSS, the effect of alkaline treatment was verified in combination with a matrix modification, specifically a reactive graft copolymerization of BioPE with maleic anhydride was considered.

## 2. Results

**Figure 1:**
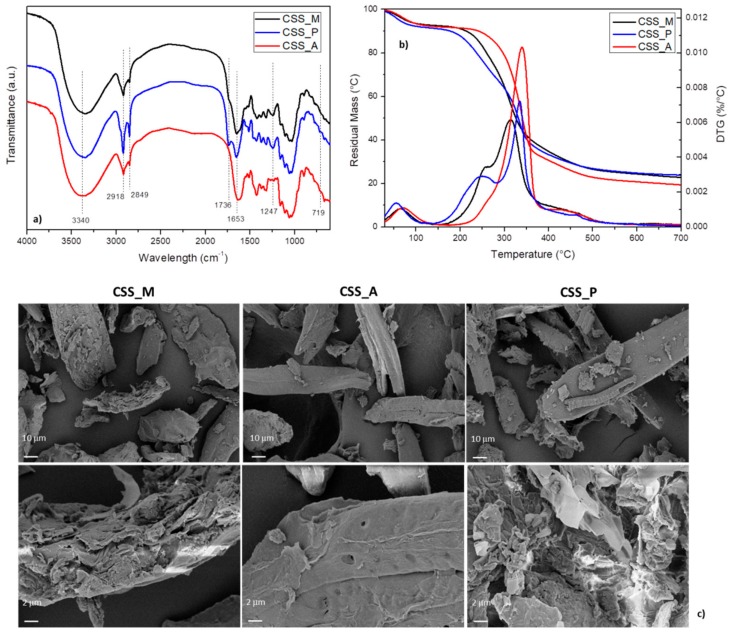
FTIR spectra (**a**) TG/DTG curves (**b**) and FESEM images (**c**) of untreated (CSS_M), alkali treated (CSS_A) and palmitoyl chloride treated (CSS_P) coffee silverskin.

The FTIR spectra of CSS, shown in Figure 1a, exhibited the typical absorption bands of lignocellulosic materials, as already reported in [29]. The broad band centered at 3340 cm^−1^ is characteristic of O-H stretching for the hydroxyl groups in polysaccharide chains. The two peaks at 2918 and 2849 cm^−1^ can be assigned to the asymmetric and symmetric stretching of CH bonds.

In the case of untreated CSS, additional bands at 1742 cm^−1^, that correspond to acetyl and uronic ester groups of hemicelluloses and the ester linkages of carboxylic group of the ferulic and *p*-coumaric acids of lignin, disappeared in CSS_A. Compared to untreated CSS, the peak at 1247 cm^−1^, that corresponds to the C–O stretching of acetyl groups of lignin, was completely removed in CSS_A. Also the hemicelluloses group was partially removed from the surface after the NaOH treatment, as evidenced by the decreased carbonyl peak at 1653 cm^−1^ [32]. The FTIR spectrum of CSS_P provides evidence that chemical bonding occurred between CSS hydroxyl groups and palmitoyl chloride. One of the major differences between the spectra of unmodified CSS and CSS_P is the appearance of ester carboxyl signal at 1736 cm^−1^, together with the intensity decrease of the hydroxyl parts. This indicates that palmitoyl groups substituted the hydrogen atoms of hydroxyl groups in CSS to form covalent ester bonds, so the increase in the intensity of absorption bands at 2918 and 2849 cm^−1^ in CSS_P can be attributed to the CH_3_ and CH_2_ vibrations in palmitoyl chloride [33]. Palmitoyl chloride grafting also resulted in the appearance of a new band around 719 cm^−1^ ascribed to CH_2_ vibrations of aliphatic chains [34].

The mass loss and derivative curves of different CSS samples, reported in Figure 1b, confirm the presence of a multistep degradation process. The first stage occurs at low temperatures (up to 110 °C) and it is mainly due to the moisture and bound water. The second step, divided into a main peak and a shoulder, occurs between 170 °C and 370 °C and it is caused by thermal decomposition of hemicellulose and lignin components. The third decomposition phase, above 370 °C, is related to slow degradation and pyrolysis of lignin and proteins. It is evident that the shoulder peak in CSS_A is not present, confirming the result of FTIR investigation, with alkali partial removal of hemicellulose and lignin components [35]. Moreover, the peak related to cellulose resulted more intense and shifted towards higher temperatures (340 °C), due to the increased amount in more thermally stable cellulose component in CSS [36,37]. CSS_P showed an improvement in thermal stability for cellulose component, with a temperature increase of the main peak (335 °C), substantially confirming the possible use of CSS_P during a thermally degrading event, such as melt extrusion [38]. However, a slight reduction of the thermal stability was induced by esterification, with an earlier thermal degradation event in the case of grafted CSS, which could be thus attributed to the limited stability of ester bonds. Residual mass values at the end of the test (700 °C) were also measured and the results indicated that, while a similar value was found for CSS_M and CSS_P (respectively found as 23% and 24%), the alkali treatment visibly increased the weight loss (up to 19%), substantially due to a faster decomposition rate of thermally unstable hemicellulose component in CSS.

FESEM images reported in Figure 1c show the morphology of natural milled CSS, in comparison with CSS_A, obtained after the treatment of soft bleaching and CSS_P, modified by esterification, at two different magnifications. The CSS_M particles show heterogeneous morphology and wide dimensional distribution from a few microns to over 100 μm, characterized by the presence of a lamellar structure, typical of vegetable teguments, with a high surface area, preferentially ordered in two dimensions. The untreated coffee silverskin fiber surface had a denser lignin-hemicellulose layer, relatively flat morphology, and very low porosity, while NaOH treatment promoted a total or partial degradation of the lignin-hemicellulose matrix, which consequently increased the surface porosity [39]. The effect of the esterification treatment of CSS_P particles is visible in different rough surface with microfibers appearance, that can correlated to the hydrophobicity of the particles [16,40].

### 2.1. Mechanical Characterization of Bulk and Thin Films Based on BioPE_CSS

**Figure 2:**
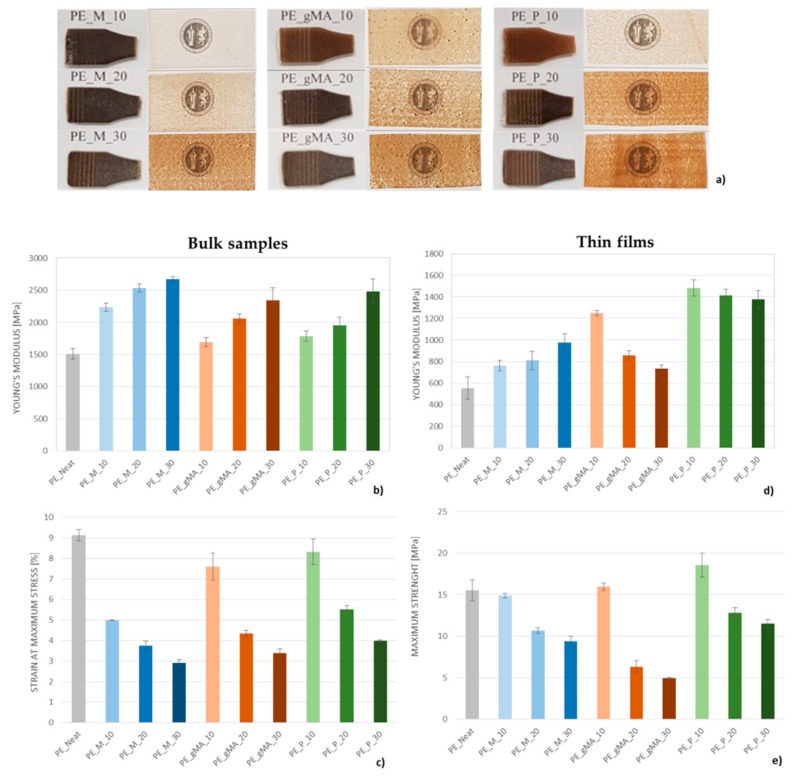
Images of bulk and thin film samples (**a**), Young Modulus and strain at maximum stress for bulk BioPE/CSS composites (**b**,**c**) and Young Modulus and strain at maximum strength for thin films BioPE/CSS composites (**d**,**e**).

Tensile characterization of bulk and thin films BioPE_CSS composites has been performed, and a summary of the results in terms of strength, elastic modulus and deformation is reported in Figure 2 and Table 1. In Figure 2a, the images of produced samples are included. When the different CSS were incorporated into the films, an evident change in color was noticed both for bulk and film samples. Specifically, color of films was changed from a very light transparent material (BioPE) to a brownish color, characteristic of the CSS filler, which results of the Maillard reaction that occur during coffee roasting process [41]. Moreover, it has been revealed that films color (Table 2) is highly dependent of the amount of CSS added to matrix, being observed a darker brown color when the concentration was increased. In general, the a* values of the films containing CSS suggested a trend to reddish, while b* values indicated a yellower appearance. L* values decreased when the CSS concentrations was higher.

Regarding the tensile characterization results, in the case of bulk samples a constant improvement in the elastic modulus and a reduction in strain at maximum stress were observed with the increase in CSS fraction for the untreated and treated CSS composites (Figure 2b,c); this phenomenon suggests that the reinforcement is much less deformable than the matrix and that it tends to delaminate at modest deformations in the tensile test. While the maximum strength remains substantially unchanged (Table 1), the strain at break was strongly reduced as the amount of reinforcement increases. Compared with the neat BioPE, all CSS-based composites showed constant tensile strength values, suggesting limited interfacial adhesion and substantially inefficient load transfer between the polymer matrix and the unmodified filler. Indeed, CSS particles acted as stress concentration points during the tensile test, resulting in a much lower elongation-at-break in polymer composites than the neat BioPE [31,42]. Overall decrease in deformation values suggests that 30wt % of CSS is a compatibility limit for filling these composites. In the case of CSS_A, alkali treatment slightly improved the strain at maximum stress, when introduced in the matrix at the lower content: mercerization creates vacancies by degrading part of the CSS surface structure, which can then be penetrated by molten PE and enhance its mechanical performance [39]. It is also evident that the use of grafted PE as compatibilizer leads to stronger interactions among particle-polymer interface, due to the fact that maleic anhydride groups can react with typical hydroxyl groups in CSS with a positive effect on deformability, as micro-crack propagation is more difficult [43]. On the other hand, hydrophobic treatment of CSS with palmitoyl chloride promotes high hydrophobicity on CSS particles which are, in turn, more compatible with the highly hydrophobic polyethylene matrix and this has a better positive effect on interface phenomena, when compared with untreated CSS, thus restricting micro-crack propagation [44].

In the case of thin films, the elastic modulus of the biocomposites films containing untreated CSS is found to be higher with respect to the BioPE matrix and higher for maximum CSS content, while a general decrease of tensile strength and elongation at break was observed: the worsening of the mechanical properties can be ascribed to the low aspect ratio of the CSS particles, which act for BioPE as a filler, and not as a reinforcement, and by the poor adhesion between CSS and the polymeric matrix. Regarding elongation at break of the biocomposites, it appears to be smaller than that of the polymeric matrix: the incorporation of the CSS filler to BioPE inhibits the deformation, which leads to reduced ductility of the material. After esterification, Young’s modulus of produced film samples was significantly improved in comparison with unmodified CSS, even if remained substantially unaltered for CSS_P containing materials at the three different weight contents. On the other hand, the E values decreased in the case of CSS_A, probably related due to a slightly detrimental treatment, that lead to surface and dimensional alteration of the CSS.

This study was intended to understand if CSS by-product could be used as a reinforcement or simply as a filler in BioPE. As a result, from the point of view of the mechanical performance, formulations containing treated silverskin show mechanical characteristics comparable and/or superior to the reference matrix.

**Figure 3:**
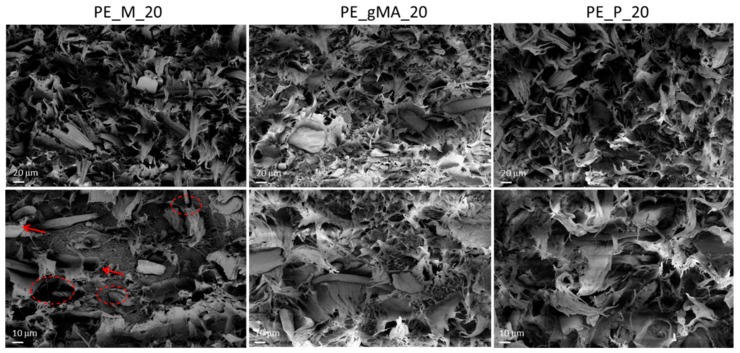
FESEM images of bulk BioPE_CSS composites with 20 wt % of unmodified, alkali and palmitoyl chloride treated CSS.

In order to investigate the morphology of the fractured surfaces, distribution of the filler and matrix/filler interface, SEM analysis was considered (Figure 3). By observing the SEM images at low magnification for bulk materials, it is evident that flat fractured surface of BioPE (not shown) is getting rough by presence of coffee silverskin. Distribution of coffee silverskin in the samples was nearly homogeneous, and no CSS clusters were found. High magnification SEM images exhibited a poor interfacial adhesion with fiber pull-out and debonding at filler/matrix interface for PE_M (red arrows and circles in magnified images): mixed mode fracture morphology, with both ductile and relatively brittle zones, was noted. In all composite materials with treated CSS (PE_gMA_20 and PE_P_20), the presence of ductile morphology prevailed and better interphase between CSS and BioPE was achieved, justifying the slight improvement already observed by mechanical characterization.

### 2.2. Wettability and Water Uptake of BioPE_CSS Films

In order to study the composites’ response to water contact, a preliminary evaluation of surface wettability was performed by means of water contact angle measurements: the reference value (74.6 ± 0.8) slightly increased from pure BioPE sample to 75.4 ± 0.9 for 10 wt % CSS_M. Afterward, by having increase in the amount of coffee silverskin in the samples, the value of contact angle was slightly increased for the two other composites, respectively to 75.5 ± 1.7 and 80.6 ± 2.1 for PE_M_20 and PE_M_30. Even if hydrophilic CSS was inserted in the film, the hydrophobicity of the film can be related to temperature processing step, which can be responsible for losing hydroxyl groups, leading to more hydrophobic composites [45]. In the case of grafted PE and use of alkali treated CSS, PE_gMA_10 showed a value of 76.1 ± 1.7, that increased to 82.1 ± 1.8 in the case of PE_gMA_30. The hydrophobic treatment registered stable values (78.2 ± 0.9 and 78.8 ± 0.7, respectively for PE_P_10 and PE_P_30). From the results which were obtained by this test, it can be noticed that even if a hydrophobic treatment was considered for the filler, film roughness and porosities which are present in the interfaces between the filler and the matrix could lead to decrease the contact angle for the composites.

**Figure 4:**
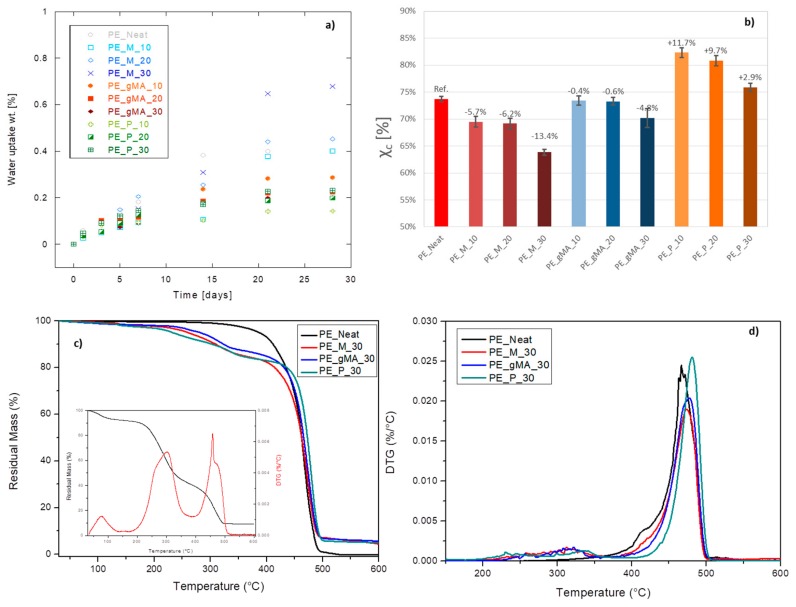
Water uptake as a function of treatment and weight amount (**a**) and crystallinity evaluation by DSC (**b**) of BioPE_CSS composites films, TG (**c**) (TG/DTG curves of unmodified CSS in the insert) and DTG (**d**) curves of neat BioPE and BioPE_CSS composites (bulk) at 30 wt % of untreated (PE_M_30), alkali treated (P_gMA_30) and palmitoyl chloride treated CSS (PE_P_30).

Water absorption tests are used to determine the amount of water absorbed by a material under specified conditions. Factors affecting the water absorption include the type of the polymer and filler which are used, volume of the filler, voids, viscosity of the matrix, humidity, temperature and length of exposure. One of the disadvantages of using natural materials such as agricultural wastes as filler in composites is the higher moisture absorption in comparison with conventional synthetic fillers, which induces dimensional changes, micro-cracking and poor thermal stability. In order to investigate the long-term application of these composites, it is necessary to study the moisture absorption and water uptake of these materials. The value of water content for all the samples initially increases with a linear trend, and then it reaches to its equilibrium amount approximately after 30 days (Figure 4a). The water absorption of all specimens was high in the early stages of exposure, and afterward it slowed down and tended to an asymptotic value at prolonged time, following a Fickian diffusion process. However, in the case of composites containing untreated CSS, increase in the filler amount leads to increase in the maximum water uptake (saturation level) of the samples: according to some research studies [46], the surface of natural fibres contains hydroxyl groups which have high affinity for water molecules. Therefore, the percentage weight gained increases as the weight fraction of CSS increases in the composite samples.

When CSS_A and CSS_P are introduced in the matrix, a substantial reduction in the water gain is obtained, mainly due to an improved hydrophobicity, as already observed by contact angle evaluations. The water absorption of composites depends on the fiber surface and the bonding between the matrix and the fiber. In PE_CSS composites, the percent gain of water absorption is initially increased with the alkali and palmitoyl chloride treatment of CSS [47,48], after that the saturation values decrease. Whether the coupling agent and surface modifier are used or not, the water uptake initially increases as a function of CSS content, after that maleated PE builds up some interactions to eliminate the amounts of OH groups and also holds hydrophobic polymer chains around CSS surface leading to less water uptake. All the samples with modified CSS have low water uptake but keep absorbing level compared to the unmodified BioPE_CSS, which takes short time to reach saturation [49]. With the presence of hydrophobized CSS, less water uptake was registered, due to the weakened absorption capacity of the functionalized CSS: this treatment seemed to be the most effective treatment to reduce water diffusion rate into the composites.

Differential calorimetric scans (not shown) evidenced no significant variations in the characteristic temperatures of composites compared to the BioPE matrix (Figure 4b). The calculation of the crystalline fraction suggests that the presence of CSS_M hinders the formation of ordered crystals by lowering the X_c_ values with increasing filler quantity. CSS_A does not produce variations on the crystalline degree, while the composites with the CSS_P show an increase of X_c_, in accordance with the trend for elastic modulus (Figure 2d). This increase progressively drops with increasing filler content, probably due to an antagonistic phenomenon between the nucleating effect of esterified fibers and the obstacle to the growth of the crystalline phase by the fibers themselves.

Results of TGA tests (Figure 4c,d) in an oxidative environment on composites containing the higher amount (30 wt %) of untreated and modified CSS revealed how the presence of thermally degradable CSS reduced the overall thermal stability of the produced composites at the lower temperature range, essentially due to the limited thermal stability of the CSS filler itself in air (see insert in Figure 4c). The T_10%_ values for PE_Neat, PE_M_30, PE_gMA_30 and PE_P_30 have been found, respectively, at 412, 310, 323 and 303 °C, showing how the introduction of the filler strongly altered the thermal behavior of the BioPE. On the other hand, temperature of main degradation peak was slightly delayed from 466 to 474, 478 and 481 °C, respectively, for PE_Neat, PE_M_30, PE_gMA_30 and PE_P_30, due to the presence of CSS as source of natural antioxidants, that contributed to both extend the initial degradation beyond 350 °C and hinder the degradation rate for all the composites.

## 3. Discussion

The aim of this study was to evaluate the application of BioPE-based composites containing up to a 30wt % of waste coffee silverskin deriving from coffee production. The composites were formulated in three different proportions (10 wt %, 20 wt %, and 30 wt %) and two compatibilizing approaches have been studied. Increasing the amount of waste in the composites, the values of elongation at tensile strength were decreased, especially for the sample with 30 wt % of CSS, in comparison with the neat matrix, while a substantial invariability of tensile strength was measured both for bulk and film samples. It was also evident that the use of grafted PE as compatibilizer and hydrophobic treatment of CSS with palmitoyl chloride has a positive effect on interface phenomena, slightly positively altering the strain at break values. The main difference between bulk and film samples was found for the elastic response of the material, due to the microsized dimension of the CSS, that actually worked as a defect in BioPE films. Studying the DSC test results, it can be understood that there were visible changes in the final crystallinity of the composites, even for the samples with 30wt % waste. Although the crystallization values for the samples decreased with respect of CSS_A containing samples, higher level of crystallinity for the CSS_P composites was measured: the compatibilizer induced the crystallization of the materials, which was reflected in limited water absorption for all the palmitoyl treated CSS composites. It can be concluded that in the present condition, the amount of filler should be limited up to 20wt %, which can result in an appropriate balance between the values of tensile strength and Young’s modulus, leading to an improve in the mechanical properties, as well as generating acceptable results in other characteristics. Furthermore, using coffee waste as filler in the production of green composites, in addition to adding value to agricultural waste, leads to a decrease in the price of renewable polymer as well as minimizing the dependency on petro-based polymers.

## 4. Materials and Methods

A high-density polyethylene (HDPE, grade SHA7260, Braskem, São Paulo, Brazil) was used as matrix. The selected HDPE grade was characterized by a minimum bio-based content of 94%, evaluated in accordance with ASTM 6866. The residue of coffee processing, consisting almost exclusively of the epidermis known as coffee silverskin (CSS), has been kindly supplied by the Tiziancaffè roasting plant in Terni, Italy. Pyridine, maleic anhydride (MA) and dicumyl peroxide (DCP) were provided by Sigma Aldrich (Milan, Italy).

### 4.1. Surface Treatments of Coffee Silver Skin

The CSS was ground in a laboratory mill (SK 100 Cross Beater, Retsch Haan, Germany) and sieved to obtain a powder with a diameter of less than 63 μm. Part of the milled CSS was named CSS Milled (CSS_M) and used without further modification. Two different treatments on the natural CSS powder to be used for the production of composites were considered: the first treatment was made by considering an alkaline treatment on the CSS in order to make the filler surface more compatible with the matrix. A soft bleaching was carried out by dipping 10 g of CSS in 1000 mL of a 0.5 M NaOH solution and stirred at 80 °C for 2 h [50]. A 0.1 M HCl solution was then added progressively to complete neutralization of the solution (pH 7.0). Subsequently, several washing steps were carried out with water to eliminate the undesired reaction products, such as NaCl salts. The CSS after the alkaline treatment (CSS_A) was dried at 50 °C for 24 h before being used to produce the composites. An esterification process with palmitoyl chloride was also considered to obtain hydrophobization of CSS. After drying at 50 °C for 24 h, 10 g of coffee silverskin powder were immersed in a 1,2-dichloroethane solution and kept in an inert atmosphere. Subsequently, palmitoyl chloride and equimolar amount of pyridine were added and the solution was kept under stirring for 2 h at 70 °C. At the end, the treated CSS was collected by filtration, washed firstly with 1,2-dichloroethane and then with water. Finally, the palmitoyl chloride treated CSS (CSS_P) was dried for 24 h at 50 °C [16,51].

### 4.2. Manufacturing of BioPE_CSS Composites

Composite materials were produced using a melt compounding method. An Xplore 15 MC co-rotating screw extruder produced by Xplore Instruments BV (Geleen, The Netherlands), with compounding function, was used. In order to make a fine comparison of obtained results, suitable process parameters (temperature profile 150–160–170 °C, compounding time = 120 s, at 50 rpm) have been kept constant for all formulations [30]. The produced formulations are shown in Table 3.

The PE_M composites were produced by directly mixing the suitable amount of milled CSS with the polymer matrix in molten phase. In the case of alkali treated CSS (CSS_A), PE_gMA composites were produced by performing a reactive extrusion of BioPE in presence of 5 wt % of maleic anhydride (MA) with respect to the matrix and 1 wt % of dicumyl peroxide (DCP) as reaction initiator. As already verified in literature, the combined use of an alkaline treatment and maleic anhydride grafting in polyolefins aims to improve the compatibility between filler and matrix [52]. PE_P composites were produced by mixing CSS Palmitoyl with the BioPE matrix without additional additives. These formulations were then used for the preparation of bulk and film samples. In detail, injected samples were produced by using a 10 CC Micro Injection Molding Machine by DSM, while the use of the extruder coupled with DSM film cast line CFL35 allowed to produce films of about 90 μm in thickness.

### 4.3. Thermal and Spectroscopic Characterization of Coffee Silver Skin

Thermal characterization by thermogravimetric analysis of untreated and surface treated CSS was performed by using an Exstar 6300 analyzer (Seiko, Tokyo, Japan) in a temperature range from 25 °C to 700 °C. Samples of 7–10 mg size were placed in standard alumina crucibles and heated at a constant rate of 10 °C/min in nitrogen atmosphere. Fourier transform infrared (FTIR) spectra were recorded on a JASCO 680 plus FTIR spectrometer (Jasco, Easton, MD, USA), by using a KBr-pellet method in the range of 4000–400 cm^−1^ wavenumbers.

### 4.4. Thermal and Mechanical Characterization of BioPE_CSS Bulk and Thin Films Composites

The tensile properties of bulk and thin film samples were obtained by using a LR30 K analyzer (Lloyd Instruments, Sussex, UK) 500 N load cell for testing of dumbbell samples, 50 N cell for the films). All samples were conditioned for 48 h at 23 ± 2 °C and at 50% of relative humidity before the test and then tested under the same conditions. According to the UNI ISO 527 test method, the tensile properties of at least 5 dumbbell samples were measured with a crosshead speed of 5 mm/min. In order to characterize the produced composites, differential scanning calorimeter (DSC) tests were also performed with Q200 DSC by TA Instruments (New Castle, Germany), in the temperature range from 25 to 210 °C, at 10 °C/min, carrying out two heating and one cooling scans in a nitrogen flow of 50 mL/min. The melting temperature (T_m_) and the crystallization temperature (T_c_) were detected as the peak temperature, respectively of the endothermic and exothermic events. The degree of crystallinity X_c_ was calculated in percentage (Equation (1)) as follows:

(1) $$\chi_{c}=100*\left[\frac{{\Delta H}_{m}}{{\Delta H}_{0}*\left(1-m_{f}\right)}\right]$$

where ΔH_*m*_ is the enthalpy for melting and ΔH_0_ is enthalpy of melting for a 100% crystalline HDPE, taken as 293 J/g [53] and (1 − m*_f_*) is the weight fraction of BioPE in the sample.

In order to evaluate the effect of CSS addition on the thermal stability of BioPE matrix and composites with 30 wt % of unmodified and treated CSS, TGA (Seiko Exstar 6300) experiments were performed from 30 to 600 °C at 10 °C min^−1^ under air atmosphere (50 mL min^−1^). Temperatures corresponding to T_10_ (temperature for 10% weight loss) and T_max_ (temperature for maximum weight loss) were measured to assess the thermal stability of the composites.

### 4.5. Water Uptake of BioPE_CSS Films

Water absorption was determined following the recommendations of the ASTM D 570 standard. Before starting the test, all the samples were dried in an oven for 24 h at 50 ± 3°, cooled in a desiccator, and immediately weighed to the nearest 0.001 g. The conditioned specimens were entirely immersed in a container of distilled water maintained at a temperature of 23 ± 1 °C for a period of 28 days and, at periodic intervals, the samples were removed from the water, wiped off with a dry cloth and immediately weighed to determine the water absorbed. The water uptake in wt % (mean value of three tested samples) was calculated according to Equation (2):

(2) $$u_{w}=100*\left[\frac{m_{w}-m_{d}}{m_{d}}\right]$$

where m*_w_* is the final wet weight after a certain immersion period and m_*d*_ is the initial dry weight of the sample before immersion. In order to better understand the response of produced composites when in contact with the water, contact angle (WCA) wettability measurements were carried up (Easydrop Standard goniometer model FM140, Kruss Hamburg, Germany) by using the Drop Shape Analysis SW21 software.

### 4.6. Colorimetry of BioPE_CSS Films

Coordinates of CIELAB colour space (L*, a*, b*) of BioPE_CSS films were determined by using a spectrophotometer CM-2300d by Konica Minolta (Milan, Italy) following CIE 15 recommendations, using D65 illuminant and 10° observer. The threshold of perception of color difference for the human eye depends on the absolute value of ΔE*_ab_ and, in a different way, from the direction of displacement along the coordinates L*, a* and b*. Generally, the color difference between two stimuli is considered imperceptible to a human observer if ΔE*_ab_ is inferior to 1 [54].

Colour difference ΔE*_ab_ between two colour stimuli (control white and sample) was calculated as the Euclidean distance between the points representing them in the space with the following Equation (3):

(3) $$\Delta E_{ab}^{*}={\left[{\left(\Delta L^{*}\right)}^{2}+{\left(\Delta a^{*}\right)}^{2}+{\left(\Delta b^{*}\right)}^{2}\right]}^{1/2}$$

### 4.7. Morphology of the of Fractured Surfaces of BioPE_CSS Bulk Composites

The morphology of the fractured surfaces was studied using a field emission scanning electron microscope (FESEM, Supra 24 by Zeiss, Oberkochen, Germany), operating at 5 kV (manufacturer, city and country). Cross-sections of both dumbbells and films were cryo-fractured by immersion in liquid nitrogen and then gold sputtered before the observation.

## Figures and Tables

**Table 1:** Results of tensile test for CSS_M, CSS_A and CSS_P BioPE composites, bulk and films samples.

Bulk Samples			Films Samples	
Sample	Maximum Strength [MPa]	Strain at Break [%]	Strain at Maximum Stress [%]	Strain at Break [%]
PE_Neat	21.1 ± 0.5	708.3 ± 0.5	13.2 ± 0.5	84.0 ± 5.5
PE_M_10	21.2 ± 1.2	25.7 ± 1.1	5.1 ± 0.7	13.7 ± 1.1
PE_M_20	22.0 ± 1.0	5.5 ± 0.3	3.3 ± 0.8	9.5 ± 0.4
PE_M_30	21.7 ± 1.8	5.0 ± 0.4	1.6 ± 0.1	3.9 ± 0.6
PE_gMA_10	21.9 ± 1.1	50.4 ± 16.5	1.8 ± 0.1	1.9 ± 0.1
PE_gMA_20	22.7 ± 0.8	8.8 ± 1.0	1.2 ± 0.4	1.8 ± 0.8
PE_gMA_30	22.8 ± 1.3	4.1 ± 0.5	0.4 ± 0.2	0.9 ± 0.2
PE_P_10	21.2 ± 1.2	52.3 ± 11.6	2.1 ± 0.4	3.1 ± 0.4
PE_P_20	20.4 ± 1.0	13.6 ± 0.4	1.9 ± 0.2	3.0 ± 0.2
PE_P_30	20.3 ± 0.8	6.9 ± 0.7	1.9 ± 0.2	2.8 ± 0.2

**Table 2:** Colour coordinates of BioPE_CSS films.

Formulations	L*	a*	b*	∆E*
White Control	99.47 ± 0.00	−0.08 ± 0.01	−0.08 ± 0.01	-
PE_Neat	99.22 ± 0.03	−0.14 ± 0.02	0.05 ± 0.02	0.26 ± 0.04
PE_M_10	93.57 ± 0.73	0.75 ± 0.11	7.55 ± 0.20	9.70 ± 0.36
PE_M_20	86.08 ± 2.10	2.55 ± 0.53	15.07 ± 1.96	20.39 ± 2.90
PE_M_30	71.54 ± 4.16	6.31 ± 1.09	23.97 ± 3.18	37.41 ± 5.33
PE_gMA_10	69.67 ± 0.96	5.93 ± 0.37	28.78 ± 1.22	41.92 ± 1.50
PE_gMA_20	68.12 ± 2.36	6.24 ± 0.58	27.38 ± 1.17	42.16 ± 2.57
PE_gMA_30	64.25 ± 5.97	7.12 ± 1.48	28.57 ± 3.09	45.99 ± 6.72
PE_P_10	71.30 ± 2.55	7.93 ± 0.89	28.97 ± 1.77	41.25 ± 3.16
PE_P_20	68.16 ± 3.66	8.90 ± 1.30	28.37 ± 3.32	43.25 ± 5.10
PE_P_30	67.76 ± 0.60	9.02 ± 0.19	27.46 ± 0.33	42.98 ± 0.69

**Table 3:** Formulated composites materials.

Matrix (wt %)	Reinforcement (wt %)	Code
100	-	PE_Neat
90	10_Milled CSS	PE_M_10
80	20_Milled CSS	PE_M_20
70	30_Milled CSS	PE_M_30
90 (MA grafted bioPE)	10_Alkali treated CSS	PE_gMA_10
80 (MA grafted bioPE)	20_Alkali treated CSS	PE_gMA_20
70 (MA grafted bioPE)	30_Alkali treated CSS	PE_gMA_30
90	10_Palmitoyl treated CSS	PE_P_10
80	20_Palmitoyl treated CSS	PE_P_20
70	30_Palmitoyl treated CSS	PE_P_30
